# Science PhD Career Preferences: Levels, Changes, and Advisor Encouragement

**DOI:** 10.1371/journal.pone.0036307

**Published:** 2012-05-02

**Authors:** Henry Sauermann, Michael Roach

**Affiliations:** 1 Georgia Institute of Technology, Atlanta, Georgia, United States of America; 2 University of North Carolina, Chapel Hill, North Carolina, United States of America; Northwestern University, United States of America

## Abstract

Even though academic research is often viewed as the preferred career path for PhD trained scientists, most U.S. graduates enter careers in industry, government, or “alternative careers.” There has been a growing concern that these career patterns reflect fundamental imbalances between the supply of scientists seeking academic positions and the availability of such positions. However, while government statistics provide insights into realized career transitions, there is little systematic data on scientists' career preferences and thus on the degree to which there is a mismatch between observed career paths and scientists' preferences. Moreover, we lack systematic evidence whether career preferences adjust over the course of the PhD training and to what extent advisors exacerbate imbalances by encouraging their students to pursue academic positions. Based on a national survey of PhD students at tier-one U.S. institutions, we provide insights into the career preferences of junior scientists across the life sciences, physics, and chemistry. We also show that the attractiveness of academic careers decreases significantly over the course of the PhD program, despite the fact that advisors strongly encourage academic careers over non-academic careers. Our data provide an empirical basis for common concerns regarding labor market imbalances. Our results also suggest the need for mechanisms that provide PhD applicants with information that allows them to carefully weigh the costs and benefits of pursuing a PhD, as well as for mechanisms that complement the job market advice advisors give to their current students.

## Introduction

Policy makers, scholars, and members of the science community are concerned that PhD-trained scientists face a shortage in available faculty positions, which are assumed to be the most desired careers in many fields [1]–[4]. Consistent with that concern, many scientists enter careers outside of academia. For example, a recent analysis of data from the 2006 Survey of Earned Doctorates conducted by the National Science Foundation shows that 5–6 years after graduation, only about 14% of PhDs in the biological sciences held tenure-track positions, compared to 21% of physicists and 23% of chemistry PhDs. Larger numbers of individuals hold non-tenure track academic positions, especially in the biological sciences (34%) and in physics (20%). Industry employs about 23% of biological scientists, 34% of physicists, and 46% of chemists 5–6 years after they had obtained their PhD [5]. Unfortunately, these aggregate numbers reflect the joint effects of both supply and demand conditions. There is little recent data on scientists' underlying career preferences and thus on the degree to which there is a mismatch between scientists' desired careers and the career opportunities actually available to them [6]. In addition, it has been suggested that career preferences may change over the course of graduate training, yet empirical evidence on such changes is limited [6], [7]. Finally, while it is sometimes argued that advisors exacerbate labor market imbalances by encouraging students to pursue faculty careers [5], [8], there is no systematic data on the degree to which advisors indeed encourage faculty versus alternative career paths. Empirical insights regarding these issues are of interest to policy makers who invest significant funds in graduate education [9], as well as to academic administrators and advisors who design graduate courses and training experiences [10], [11]. Perhaps most importantly, such insights may also help junior scientists in thinking about their future career paths.

In this paper we draw on novel survey data to provide unique insights into PhD students' career preferences, changes in preferences over the course of the PhD program, and faculty advisors' encouragement of specific career paths. In conjunction with existing data on the realities of labor market opportunities, our results speak to common concerns regarding labor market imbalances. At the same time, our data suggest the need to consider important differences across fields.

## Results

We conducted a large-scale survey among PhD students at 39 tier-one U.S. research universities in the spring of 2010. Our sample includes 4,109 PhD students in the life sciences (59%), chemistry (18%), and physics (23%). Table S1 shows a complete listing of universities included in the sample and Table S2 provides a listing of subfields. Thirty-six percent of respondents indicated that they were on the job market at the time of the survey or were planning to be on the job market within the next year, and 26% of respondents had not yet completed their qualifying exam or similar milestones. The average time in the program was 3.7 years. The Materials and Methods section below provides a detailed discussion of the survey. Table S3 shows summary statistics.

Our empirical analysis proceeds as follows. First, we describe the measures of career preferences and provide insights into the levels of students' preferences for careers in academia (faculty research and faculty teaching), industry (established firms and startups), as well as government R&D and “other” careers. We then examine changes over time by comparing preferences across cohorts of students and by comparing current and retrospective measures within a given student. Third, we provide data on the degree to which students perceive that their advisors or departments encourage or discourage particular careers. Finally, we provide detailed insights into respondents' interests in particular work activities such as basic research, applied research, or technology commercialization.

### Levels of career preferences

**Figure 1:**
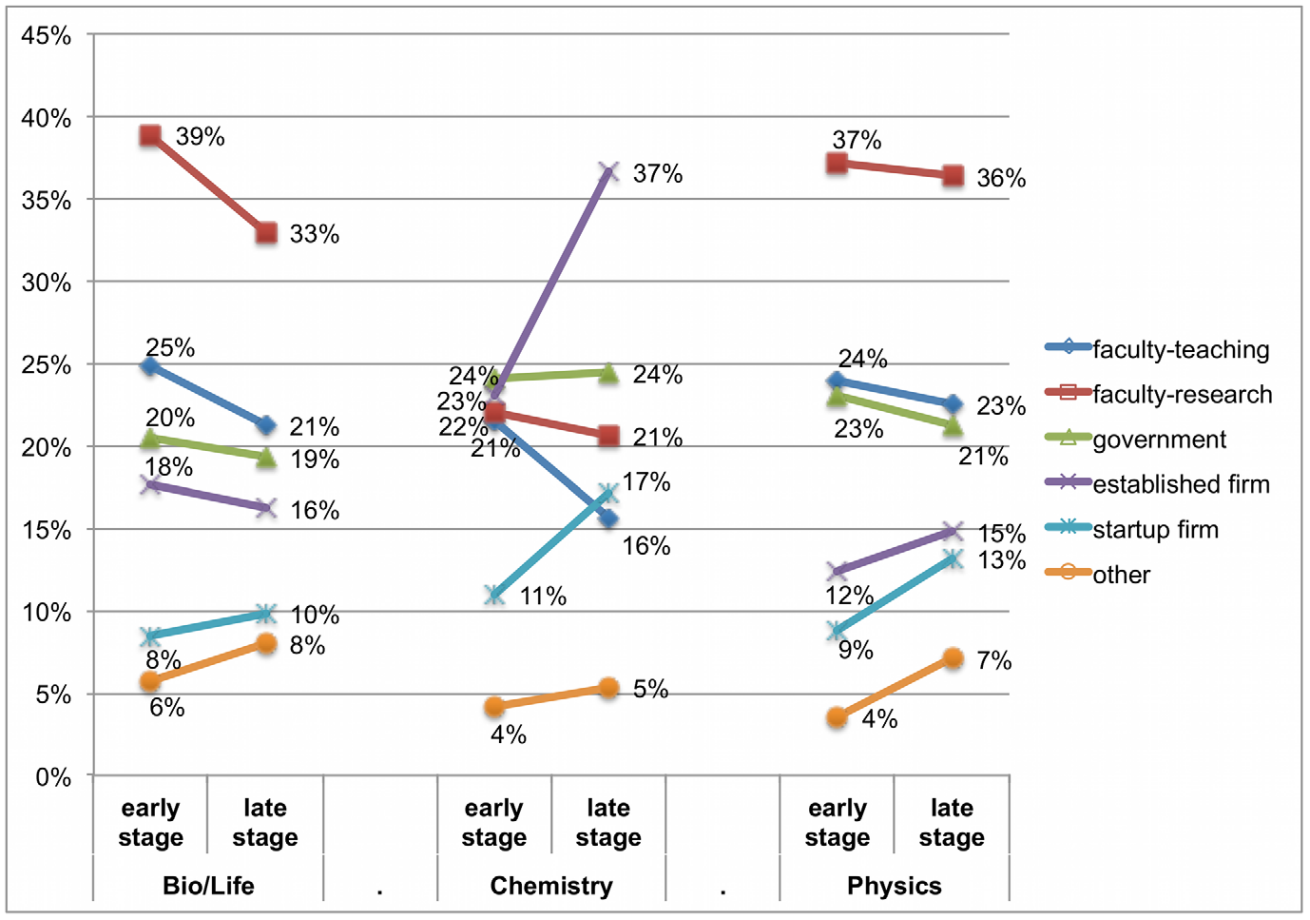
Students judging a career “extremely attractive” by field and stage in program. Respondents rated the attractiveness of each career on a 5-point scale (and were instructed to ignore job availability). The scale anchors ranged from 1 (extremely unattractive) to 3 (neither attractive nor unattractive) to 5 (extremely attractive). Figure 1 shows the share of respondents who gave a rating of 5 (“extremely attractive”) to a particular career. Data are shown separately for respondents in the early stages of the PhD program (prior to completion of qualifying exams or similar milestones) and in the late stages of the PhD program (looking for a job at the time of the survey or planning to do so within the next year).

Our primary interest is in respondents' career preferences, i.e., which career paths they find attractive regardless of job market conditions. Thus, we asked respondents to ignore job availability and rate how attractive they find each of the following careers: (a) a faculty career with an emphasis on teaching; (b) a faculty career with an emphasis on research or development; (c) a government job with an emphasis on research or development; (d) a job in an established firm with an emphasis on research or development; (e) a job in a startup with an emphasis on research or development; and (f) other career. Since additional postdoctoral training is very common in some fields [12], [13], we explicitly asked respondents to state their career preferences with respect to employment after graduation and any potential postdocs. Table S4 provides detailed data on the distribution of responses in each response category, ranging from 1 (“extremely unattractive”) to 5 (“extremely attractive”). Figure 1 shows the percentage of respondents rating a particular career as extremely attractive (score of 5) by broadly defined field. Figure 1 shows results separately for students in early stages of the PhD program and for those who were on the job market in the year of the survey or were planning to look for jobs within the next year.

Consistent with field differences in actual career patterns [5], we observe considerable differences in career preferences across fields. Across all cohorts, students in the life sciences and physics most often rate a faculty career with an emphasis on research as extremely attractive (34% and 38% of students, respectively), followed by teaching careers and R&D positions in government. Among chemistry PhD students, an R&D career in an established firm is most often considered extremely attractive (27%), followed by R&D careers in government (21%). Figure 1 also shows that some respondents find “other” career extremely attractive. We asked respondents to specify which particular career they were thinking of, and the most commonly mentioned careers include science communication/writer, science policy, non-university teaching, working for a non-profit/NGO, and consulting.

**Figure 2:**
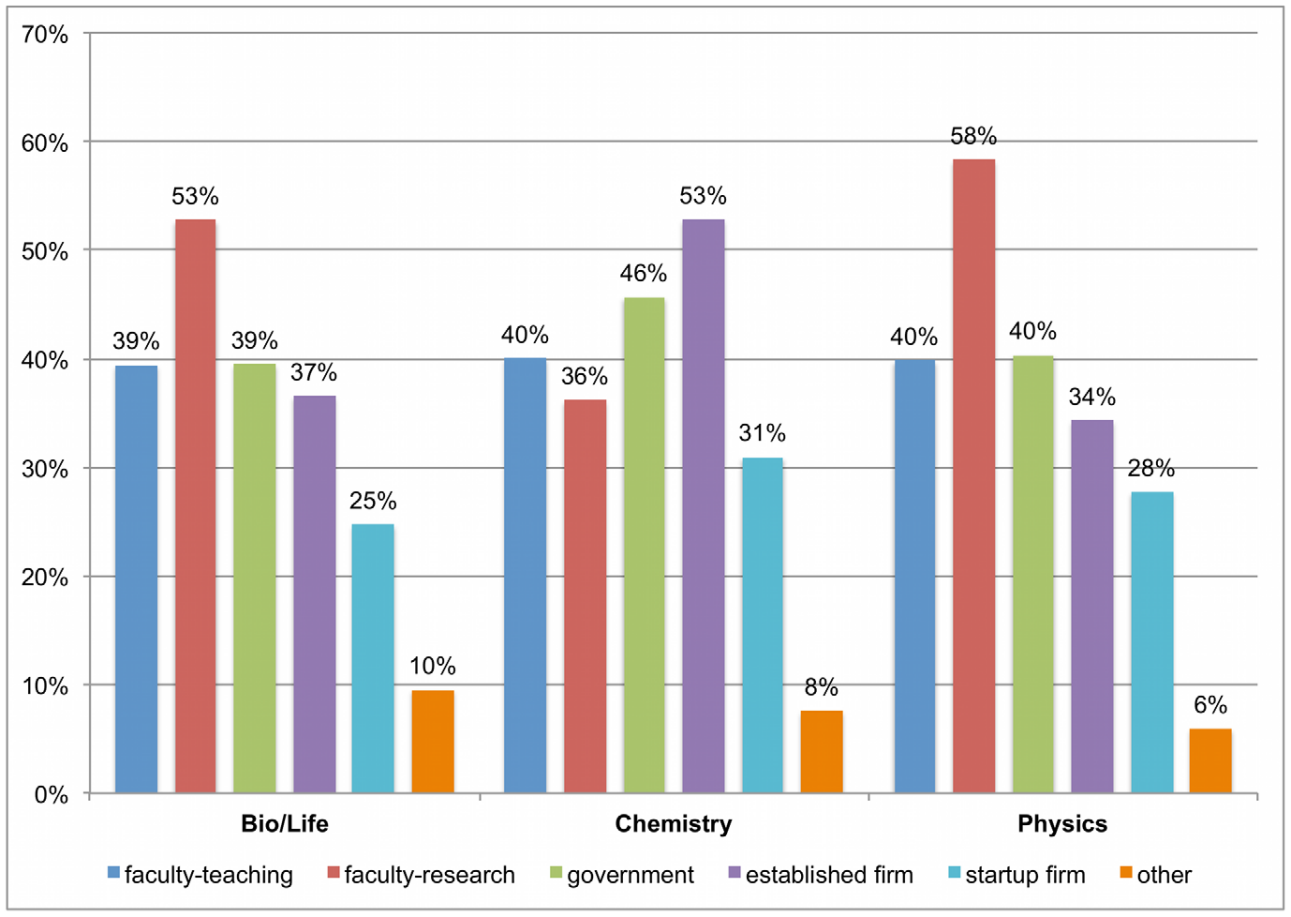
Most attractive career path (full sample; ties possible). Respondents rated the attractiveness of each career path on a 5-point scale. Figure 2 shows the share of respondents who gave their highest rating to a particular career. For example, 53% of life sciences PhD students gave their highest attractiveness rating to the faculty research career. Since careers were rated independently, careers can be tied (i.e., receive the same attractiveness score).

Figure 1 shows the share of students who find a particular career extremely attractive in an absolute sense. To assess the attractiveness of the various career paths *relative to each other*, we coded a new set of variables, indicating which of the six career options received the highest attractiveness rating. Since respondents may judge multiple careers as similarly attractive, this measure also includes ties. Figure 2 shows that a faculty position with focus on research is among the most attractive careers for over 50% of life scientists and physicists, while a research position in an established firm is among the most attractive options for over 50% of chemists.

### Changes over time

In addition to important differences across fields, Figure 1 also shows significant differences across cohorts of students within a given field. For example, the share of life sciences students finding a faculty research career extremely attractive is significantly lower in the late stage versus the early stage of the PhD program (33% vs. 39%, p<0.01). Similarly, the share of life sciences students finding a faculty teaching career extremely attractive declines from 25% to 21% (p<0.05). In chemistry, we observe a significant decrease in the share of students finding teaching careers extremely attractive (21% vs. 16%, p<0.01) and a sharp increase in the attractiveness of careers in industry (37% vs. 23%, p<0.01). There is some evidence that the attractiveness of startup careers increases in all three fields, although these changes are not statistically significant at conventional levels of confidence.

Decreases in the attractiveness of faculty careers and concomitant increases in the attractiveness of nonacademic careers lead to even sharper shifts in the share of students finding a particular career *most* attractive compared to all other careers (the measure used in Figure 2). In particular, the share of students finding a faculty research career most attractive drops in all three fields, from 57% for the early cohort to 50% for the late cohort in the life sciences, from 45% to 32% in chemistry, and from 60% to 53% in physics.

The detailed data presented in Table S4 show changes not only in the share of students who find particular careers extremely attractive, but also in the share of students who find particular careers unattractive. Most notably, we find that the share of students who find a faculty research career “unattractive” or “extremely unattractive” increases from 11% to 21% (p<0.01) in the life sciences, 22% to 38% (p<0.01) in chemistry, and 7% to 14% (p<0.05) in physics.

One interpretation of these differences across cohorts is that students' preferences change over the course of graduate training. For example, students may enter graduate school with overly positive views of the faculty career and may change their expectations upon experiencing academic life first-hand [7], [14]–[16]. Similarly, students may learn about career paths outside of academia and may come to appreciate their advantages [7], [17]. Moreover, even though our question asked students to ignore job availability, the responses of some later-stage students may reflect that they realized over time that they are not competitive for scarce academic jobs and thus ceased to “want” them.

**Figure 3:**
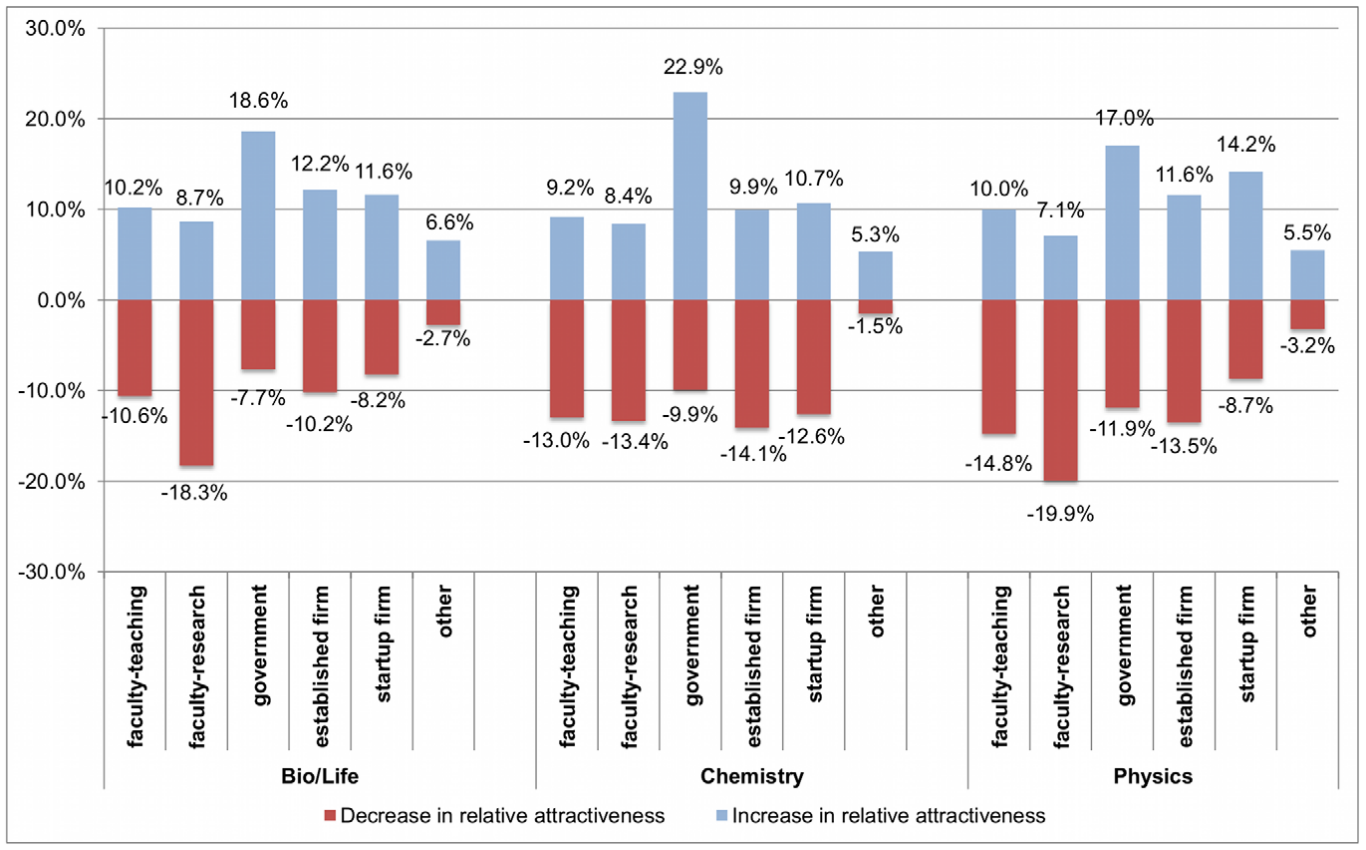
Change in the relative attractiveness of careers over time (respondents in the late stage of the PhD). Respondents were asked how certain they were at the time of beginning the PhD program to pursue each career. Similarly, respondents were asked how attractive they found each career at the time of the survey. For each of the two points in time, we coded which career received the highest rating (ties possible). Positive numbers in Figure 3 show the share of respondents who gave the highest rating to a particular career at the time of the survey but not when starting the PhD (i.e., the relative attractiveness of that particular career increased). Negative numbers show the share of respondents who gave the highest rating when starting the PhD but not at the time of the survey (i.e., the relative attractiveness decreased). For example, the relative attractiveness of a faculty research career increased over the course of the program for 8.7% of life sciences PhD students but decreased for 18.3% of life sciences PhD students. The net effect is a decrease in the share of students who rate the faculty career as most attractive by 9.6 percentage points.

In addition to such changes within a given individual, however, the differences across cohorts reported in Figure 1 may also reflect “cohort effects” [18]. More specifically, the students who were in the late stage of the PhD at the time of the survey may have been different from those in the early stage even when they initially entered the PhD program, e.g., due to different labor market conditions at the time of enrollment in the PhD. To more clearly assess changes over time for a given individual and to eliminate cohort effects, we asked respondents in the late stage of the PhD in what year they started their program and to recall how certain they were at that time to pursue the various career options. We examined changes in career preferences within a given individual by comparing which career received the highest rating at the time of the survey versus at the time of enrollment in the PhD program. Figure 3 visualizes these changes over time. For example, Figure 3 shows that 18.3% of respondents in the life sciences rated a faculty research career highest when starting their PhD program, but did not rate this career highest at the time of the survey. Thus, relative to other careers, the faculty research career became less attractive for 18.3% of life sciences PhD students. At the same time, 8.7% of them rated the faculty research career as most attractive at the time of the survey, even though they had not done so at the time of joining the PhD program; for these respondents, the faculty research career became relatively more attractive over time. Taken together, these numbers suggest an overall decline in the relative attractiveness of the faculty research career among life sciences PhD students: the share of respondents who rated this career highest declined by 9.6 ( = 18.3–8.7) percentage points. This drop is even more pronounced in physics, where the share of respondents who rated the faculty research career highest dropped by 12.8 percentage points. In chemistry, the share decreases by 5 percentage points.

If academic research became relatively less attractive over time, which careers became relatively more attractive? Figure 3 shows that many students at the end of the PhD program consider an R&D career in government the most attractive, even though they had not done so at the beginning of the PhD. More specifically, the share of respondents who rate this career highest increased by 10.9 percentage points in the life sciences, 13 percentage points in chemistry, and 5.1 percentage points in physics. While our survey itself does not provide insights into the underlying drivers of this change, informal interviews with PhD students suggest that perceived high levels of job security and access to funding, as well as the recognition that government labs provide opportunities to do quite “academic” research may play an important role. Note, however, that changes in the attractiveness of government jobs emerge only in the within-individual analysis; we did not find significant differences between early and late cohorts (see Figure 1).

Despite the decline in the attractiveness of faculty careers over time, our data show that the faculty research career remains extremely attractive to a large share of graduating students in the life sciences and in physics (see Figure 1). As detailed in the introduction, however, NSF data show that the share of graduates who are actually able to obtain tenure track faculty positions is significantly smaller [5]. Thus, our data on career preferences complement existing data on available positions and provide empirical support for growing concerns about imbalances in the scientific labor market [1], [3], [16].

### Advisor encouragement

**Figure 4:**
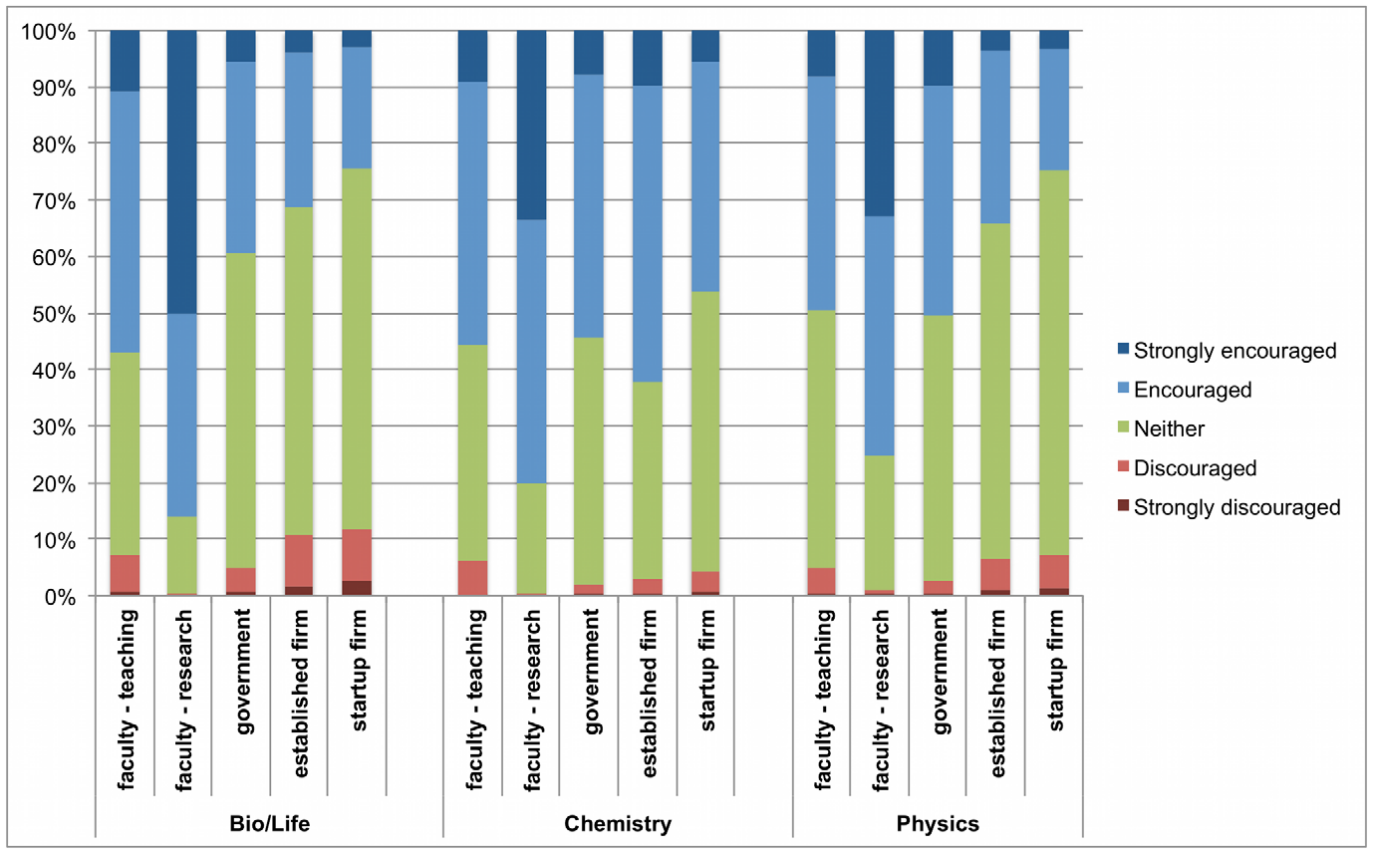
Share of students reporting that particular careers are encouraged/discouraged in their lab or department. Respondents rated on a 5-point scale the degree to which PhDs in their lab/department are encouraged or discouraged to pursue each career. Figure 4 shows the share of respondents choosing each response category. Raw data for this figure are shown in Table S5.

The strong interest in faculty research positions despite the low availability of such positions raises the question to what extent advisors and departments further encourage students to pursue academic positions and to what extent they are supportive of careers in other sectors. Despite the common belief that advisors have a strong interest in encouraging students to enter academic careers [5], [8], [19], systematic evidence is lacking. We asked respondents to what extent they felt that PhD students in their lab/department are encouraged or discouraged to pursue the various careers, using a scale ranging from 1 (strongly discouraged) to 3 (neither discouraged nor encouraged) to 5 (strongly encouraged). The results are plotted in Figure 4; the source data are shown in Table S5. Figure 4 shows that the faculty research career is indeed by far the most often “strongly encouraged” career. A small number of students feel that certain other careers are explicitly discouraged, mostly teaching careers and careers in industry. It is notable that encouragement for faculty careers and discouragement for industry careers are especially pronounced in the life sciences, where the share of graduates obtaining tenure track faculty positions is smallest and where much of the discussion around labor market imbalances takes place [5]. Even in chemistry, where industry careers are very common and where students express a strong interest in industry careers, students feel that research careers in academia are much more strongly encouraged.

Figure 4 also shows that a considerable share of students feels that non-academic careers are neither encouraged nor discouraged. One possible interpretation is that these careers are discussed between students and their advisors and that the latter explicitly take a “neutral” stance with respect to these careers. Alternatively, these career options may not be very salient in student-advisor discussions, and the neutral ratings in Figure 4 may reflect a lack of guidance and information regarding these careers rather than an explicit neutral position. Further research on the depth and scope of advisor-student discussions regarding career trajectories is needed to disentangle these two mechanisms.

### Interest in different kinds of work activities

**Figure 5:**
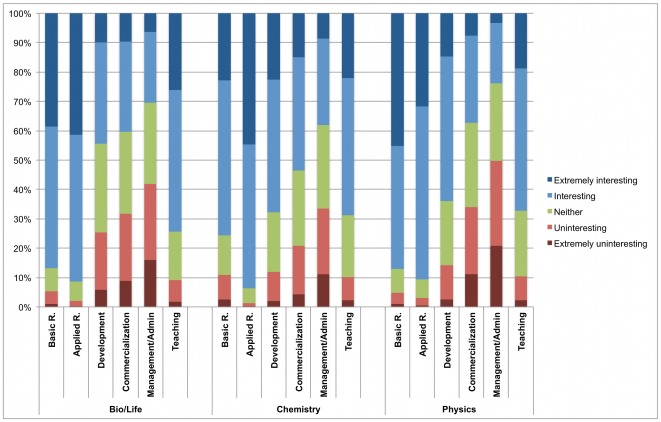
Share of students finding particular work activities interesting/uninteresting. Respondents indicated how interesting they would find each of six kinds of work when thinking about the future. Figure 5 shows the share of respondents choosing each response category. Raw data for this figure are shown in Table S6.

While our focus is on students' preferences for different types of careers and employment sectors, we also collected data specifically on their interest in different types of work. In particular, we asked respondents how interesting they would find each of 5 different types of work in the future, including “research that contributes fundamental insights or theories (basic research);” “research that creates knowledge to solve practical problems (applied research);” “using knowledge to develop materials, devices, or software (development);” “commercializing research results into products or services;” “management/administration;” and “teaching.” Figure 5 shows the distribution of ratings, ranging from “extremely uninteresting” to “extremely interesting” (source data in Table S6). In the life sciences and in chemistry, the largest share of “extremely interesting” ratings is given to applied research. Among physicists, basic research is most often rated as “extremely interesting.” Teaching is rated as “extremely interesting” by approximately 20% of respondents, with only small differences across fields.

In light of a growing interest in translational research and academic entrepreneurship in both the scholarly and the policy communities [20]–[22], it is notable that many students in the life sciences and in chemistry have a strong interest in research that solves concrete problems. At the same time, the share of scientists who would be interested in getting actively involved in technology commercialization is significantly smaller, and many respondents find commercialization uninteresting or even extremely uninteresting. Future research is needed to examine how the distribution of work interests matches with the needs of prospective employers in the various sectors of the economy.

## Discussion

Our data show that a faculty research career is the career path most often considered “extremely attractive” and ranks among the most desirable careers for over 50% of life scientists and physicists. Given that the number of faculty positions is much smaller [5], these findings support the concern that the supply of science PhDs interested in faculty research positions significantly exceeds the number of available positions in these fields. At the same time, the majority of chemistry students as well as significant shares of students in the life sciences and in physics prefer careers outside of academia, regardless of job availability. Academic administrators and advisors should consider such heterogeneity in career preferences when designing graduate curricula, ensuring that students have opportunities to acquire the skills and knowledge required to perform in non-academic careers that may not only be more readily available but are also quite attractive to students themselves [6], [10]. Similarly, the public discussion may benefit from recognizing that labor market experiences may be quite different depending on which particular career a junior scientist seeks to pursue.

Second, respondents across all three major fields feel that their advisors and departments strongly encourage academic research careers while being less encouraging of other career paths. Such strong encouragement of academic careers may be dysfunctional if it exacerbates labor market imbalances or creates stress for students who feel that their career aspirations do not live up to the expectations of their advisors. In the context of prior findings that students feel well-informed about the characteristics of academic careers but less so about careers outside of academia [17], our results suggest that PhD programs should more actively provide information and training experiences that allow students to learn about a broader range of career options, including those that are currently less encouraged. Richer information and a more neutral stance by advisors and departments will likely improve career decision-making and has the potential to simultaneously improve labor market imbalances as well as future career satisfaction [23], [24]. Advisors' apparent emphasis on encouraging academic careers does not necessarily reflect an intentional bias, however. Rather, it may reflect that advisors themselves chose an academic career and have less experience with other career options. Thus, administrators, policy makers, and professional associations may have to complement the career guidance students' advisors and departments provide.

Third, our data suggest that students' interest in academic research declines over the course of the PhD training, while other careers become relatively more attractive. Future research is needed to examine the underlying sources of such changes and potential implications for science education and scientific labor markets. The observed changes in career preferences may be beneficial if they reflect that students acquire more information about career options, potentially leading to better career decisions. However, a declining interest in a faculty research career may also imply a greater divergence between students' interests on the one hand, and the academic orientation of traditional PhD curricula as well as advisor expectations on the other [8]. To the extent that the strong interest in a faculty career at the beginning of the PhD reflects a lack of information about the challenges and job prospects of faculty careers, providing such information to applicants *prior* to enrollment in the PhD may allow them to more accurately evaluate the costs and benefits of pursuing a PhD. Of course, stronger (self−)selection prior to enrollment may reduce the number of graduate students available to work in academic labs, potentially requiring changes to how scientific labor is organized in academic research [3], [4].

This study is not without limitations. First, our sample is drawn from larger PhD programs at tier- one institutions. While the institutions in our sample account for a large share of the total production of U.S.-trained PhDs, our results may not generalize to students in smaller or lower-tier programs. Second, even though we explicitly asked students to ignore job availability, the weak job market may have led some respondents to understate the attractiveness of hard to get positions. While we believe that any such effect is small, it would imply that scientists' “true” preferences for faculty careers are even stronger than shown in the data, suggesting an even larger mismatch between career preferences and career opportunities.

## Materials and Methods

### Ethics statement

This research has been approved by the Georgia Institute of Technology's Institutional Review Board. Given the sensitive nature of the data, all respondents were ensured confidentiality. Respondents read a consent form prior to taking the survey and agreed by clicking on a link to proceed with the web survey. The data shown in this study have been anonymized.

### Data collection

We identified 39 tier-one U.S. research universities with doctoral programs in science and engineering fields by consulting the National Science Foundation's reports on earned doctorates [25]. Our selection of universities was based primarily on program size while also ensuring variation in private/public status and geographic region. The 39 universities in our sample produced roughly 40% of the graduating PhDs in S&E fields in 2009 [25]. Table S1 shows the number of cases in each of the 39 universities. While our results should be representative of students at larger tier-one universities, they do not necessarily generalize to graduate students at smaller and lower-tier institutions.

We collected roughly 30,000 individual names and email addresses from listings provided on our target departments' websites. We invited these individuals to participate in the survey using a four-contact strategy (one invitation, three reminders). All surveys were conducted online, using the software suite Qualtrics (www.qualtrics.com). Adjusting for 6.3% undeliverable emails, the direct survey approach achieved a response rate of 30%. This response rate reflects respondents who actually finished the survey, i.e., who saw all pages of the survey and pressed “next” on the final page. We dropped respondents who started the survey but did not finish it. Item non-response among those who finished was low (less than 2%) and we imputed missing items using multiple regression. Further details on the survey strategy are provided in [26].

When individual contact information was not available, we used department administrators as a second channel to approach respondents. In those cases, we emailed administrators with the request to forward a survey link to their graduate students and our research assistants additionally called administrators on the telephone to encourage their cooperation. Overall, 88% of our responses were obtained directly from respondents and 12% were obtained through administrators.

The initial survey sample is very broad and this study focuses on the sub-sample of 4,109 PhD students in the life sciences (59%), chemistry (17.7%), and physics (23.2%). According to data from the Survey of Earned Doctorates, the comparable shares of PhD degrees granted in the US in 2009 are 68% for the life sciences, 18% for chemistry, and 14% for physics [25]. We conducted all analyses separately by field such that the oversampling of physics PhDs does not affect our results. Table S2 shows the number of cases in each subfield.

### Measures

#### Current career preferences

We asked respondents: *Putting job availability aside, how attractive do you personally find each of the following careers?*

- *University faculty with an emphasis on teaching*
- *University faculty with an emphasis on research or development*
- *Government job with an emphasis on research or development*
- *Job in established firm with an emphasis on research or development*
- *Job in startup/entrepreneurial firm with an emphasis on research or development*
- *Other (please specify):*

Respondents rated each career on a 5-point scale ranging from 1 (extremely unattractive) to 3 (neither attractive nor unattractive) to 5 (extremely attractive). This item was placed in a section of the questionnaire beginning with “The following questions refer to future employment after graduation and any potential postdocs.”

#### Stage in the PhD program

We asked respondents: *What stage are you in the PhD program? Please check all that apply.*

- *have not yet passed my qualifying exam*
- *am working on my dissertation research*
- *am working on non-dissertation research (e.g., as research assistant)*
- *intend to begin actively looking for a job or post-doc position within the next year*
- *am actively looking for a job or a post-doc position*

We coded the following three dummy variables: STAGE_EARLY = 1 if a respondent checked the first option. STAGE_LATE = 1 if respondent checked one of the last two options. STAGE_MIDDLE otherwise.

Career preferences at the start of the PhD program. We asked respondents: *Thinking back to when you began your PhD program in [year], how certain were you at that time that you wanted to pursue the following careers?*

- *University faculty with an emphasis on teaching*
- *University faculty with an emphasis on research or development*
- *Government job with an emphasis on research or development*
- *Job in established firm with an emphasis on research or development*
- *Job in startup/entrepreneurial firm with an emphasis on research or development*
- *Other (please specify):*

Respondents rated each option on a 5-point scale ranging from 1 (certain not to pursue) to 3 (uncertain whether to pursue) to 5 (certain to pursue).

#### Interest in research and non-research work activities

We asked respondents: *When thinking about the future, how interesting would you find the following kinds of work?*

- *Research that contributes fundamental insights or theories (basic research)*
- *Research that creates knowledge to solve practical problems (applied research)*
- *Using knowledge to develop materials, devices, or software (development)*
- *Commercializing research results into products or services*
- *Management/Administration*
- *Teaching or training others*

Respondents rated each item on a 5-point scale ranging from 1 (extremely uninteresting) to 3 (neither interesting nor uninteresting) to 5 (extremely interesting).

#### Degree to which careers are encouraged/discouraged in lab/department

We asked respondents: *In your lab/department, to what extent are PhDs encouraged or discouraged to pursue the following careers?*

- *University faculty with an emphasis on teaching*
- *University faculty with an emphasis on research or development*
- *Government job with an emphasis on research or development*
- *Job in established firm with an emphasis on research or development*
- *Job in startup/entrepreneurial firm with an emphasis on research or development*

Respondents rated each item on a 5-point scale ranging from 1 (strongly discouraged) to 3 (neither encouraged nor discouraged) to 5 (strongly encouraged).

Subfield. We asked respondents: *Which of the following best describes your general field and area of specialization?* Respondents selected one of the options shown in Table S2. Given the framing of the question, we assume that respondents in interdisciplinary programs chose the field that best reflects their current work.

### Measurement issues

In line with prior research on S&E career preferences [11], [17], [27], we rely on direct measures of preferences by asking the decision makers. An alternative approach to measuring preferences is to infer preferences from observed choices or outcomes [28]–[30]. While both measurement approaches have their advantages, the latter “revealed preferences” approach assumes that individuals do in fact have a choice between the relevant alternatives. In our particular context, inferring career preferences from actual career transitions could underestimate scientists' preferences for academic careers if academic positions are in limited supply and some scientists who would prefer an academic position are forced to take positions in other sectors. We sought to further reduce the influence of labor market conditions by asking respondents explicitly to ignore job availability. Thus, we seek to understand which careers junior scientists find attractive rather than which careers they think they will have to pursue due to job market conditions. This aspect is particularly important given potential imbalances in scientific labor markets. While our approach may not completely eliminate the influence of job market conditions, it provides a clearer assessment of preferences than either realized career transitions or self-reports that do not ask respondents to ignore job market conditions.

A general concern with self-reported measures of preferences for careers or work activities is that respondents may overstate preferences that seem socially desirable (e.g., research in academia) and give artificially low scores to preferences that may seem less socially desirable [31]. To mitigate this concern, we stated clearly in the survey invitation that responses would be kept strictly confidential.

One of our analyses of changes over time relies on retrospective measures of career preferences at the start of the PhD program. While retrospective questions can be useful if no real-time measure is available, respondents may not always accurately report past behaviors and intentions. It has been suggested, for example, that respondents sometimes assume unrealistic high degrees of stability, resulting in retrospective reports that are more similar to current behaviors and intentions than is warranted [32], [33]. Similarly, respondents may be motivated to report past intentions that are similar to current intentions or outcomes in order to appear “consistent.” While we are not able to explicitly assess the potential for such biases in our data, both effects would suggest that our estimates of within-individual changes in career preferences (Figure 3) are conservative. Future research assessing changes in career preferences using multiple real-time measurements is needed to complement our analysis.

Finally, in interpreting the results regarding advisor encouragement, it has to be kept in mind that our measures reflect students' perceptions of the degree to which certain careers are encouraged/discouraged in their lab or department. While these perceptions should have the most direct impact on junior scientists' career decisions, future research should also examine objective measures of advisor encouragement.

## Supporting Information

Table S1 **Universities included in sample and number of cases in each.**
(DOCX)

Table S2 **Subfields and number of cases in each.**
(DOCX)

Table S3 **Summary statistics, by field.**
(DOCX)

Table S4 **Detailed distribution of current career preferences, by stage in program.**
(DOCX)

Table S5 **Data for** **Figure 4** **(share of students reporting that particular careers are encouraged/discouraged in their lab or department).**
(DOCX)

Table S6 **Data for** **Figure 5** **(share of students finding particular work activities interesting/uninteresting).**
(DOCX)
