# Supplementary material for: Science PhD Career Preferences: Levels, Changes, and Advisor Encouragement
Source: PLoS One. 2012 May 2;7(5):e36307. doi: 10.1371/journal.pone.0036307 (PMC3342243; doi:10.1371/journal.pone.0036307)
Supplement: Table S1 — Universities included in sample and number of cases in each. (DOCX) [file pone.0036307.s001.docx]

Table S1: Universities included in sample and number of cases in each

| **University** | **N** | **Percent** |
| --- | --- | --- |
| UNIVERSITY OF CALIFORNIA-BERKELEY | 246 | 5.99 |
| UNIVERSITY OF WASHINGTON | 243 | 5.91 |
| UNIVERSITY OF CALIFORNIA-DAVIS | 218 | 5.31 |
| UNIVERSITY OF WISCONSIN-MADISON | 213 | 5.18 |
| JOHNS HOPKINS UNIVERSITY | 195 | 4.75 |
| UNIVERSITY OF FLORIDA | 185 | 4.50 |
| CORNELL UNIVERSITY | 150 | 3.65 |
| UNIVERSITY OF CALIFORNIA-SAN DIEGO | 143 | 3.48 |
| UNIVERSITY OF NORTH CAROLINA AT CHAPEL HILL | 143 | 3.48 |
| MICHIGAN STATE UNIVERSITY | 142 | 3.46 |
| UNIVERSITY OF CHICAGO | 136 | 3.31 |
| DUKE UNIVERSITY | 135 | 3.29 |
| UNIVERSITY OF MICHIGAN-ANN ARBOR | 128 | 3.12 |
| YALE UNIVERSITY | 114 | 2.77 |
| WASHINGTON UNIVERSITY IN ST. LOUIS | 110 | 2.68 |
| HARVARD UNIVERSITY | 106 | 2.58 |
| EMORY UNIVERSITY | 104 | 2.53 |
| UNIVERSITY OF ILLINOIS AT URBANA-CHAMPAIGN | 96 | 2.34 |
| UNIVERSITY OF MINNESOTA-TWIN CITIES | 93 | 2.26 |
| TEXAS A & M UNIVERSITY | 92 | 2.24 |
| UNIVERSITY OF CALIFORNIA-LOS ANGELES | 89 | 2.17 |
| MASSACHUSETTS INSTITUTE OF TECHNOLOGY | 87 | 2.12 |
| OHIO STATE UNIVERSITY MAIN CAMPUS | 87 | 2.12 |
| COLUMBIA UNIVERSITY IN THE CITY OF NEW YORK | 86 | 2.09 |
| UNIVERSITY OF TEXAS AT AUSTIN | 85 | 2.07 |
| PURDUE UNIVERSITY MAIN CAMPUS | 76 | 1.85 |
| IOWA STATE UNIVERSITY | 75 | 1.83 |
| NORTH CAROLINA STATE UNIVERSITY | 73 | 1.78 |
| PRINCETON UNIVERSITY | 73 | 1.78 |
| UNIVERSITY OF MARYLAND COLLEGE PARK | 71 | 1.73 |
| PENN STATE UNIVERSITY | 68 | 1.65 |
| NORTHWESTERN UNIVERSITY | 59 | 1.44 |
| UNIVERSITY OF CALIFORNIA-IRVINE | 38 | 0.92 |
| GEORGIA INSTITUTE OF TECHNOLOGY | 37 | 0.90 |
| STANFORD UNIVERSITY | 36 | 0.88 |
| UNIVERSITY OF CALIFORNIA-SAN FRANCISCO | 27 | 0.66 |
| CALIFORNIA INSTITUTE OF TECHNOLOGY | 22 | 0.54 |
| UNIVERSITY OF SOUTHERN CALIFORNIA | 20 | 0.49 |
| RENSSELAER POLYTECHNIC INSTITUTE | 8 | 0.19 |
| **Total** | **4,109** | **100.00** |
