# Supplementary material for: Science PhD Career Preferences: Levels, Changes, and Advisor Encouragement
Source: PLoS One. 2012 May 2;7(5):e36307. doi: 10.1371/journal.pone.0036307 (PMC3342243; doi:10.1371/journal.pone.0036307)
Supplement: Table S2 — Subfields and number of cases in each. (DOCX) [file pone.0036307.s002.docx]

Table S2: Subfields and number of cases in each

| **Subfield** | **N** | **Percent** |
| --- | --- | --- |
| BIOLOGICAL/LIFE SCIENCES (general) | 184 | 4.48 |
| Biochemistry/biophysics | 416 | 10.12 |
| Cell/molecular biology | 448 | 10.90 |
| Developmental biology/embryology | 92 | 2.24 |
| Ecology | 245 | 5.96 |
| Genetics | 236 | 5.74 |
| Immunology | 137 | 3.33 |
| Microbiology | 202 | 4.92 |
| Neuroscience | 381 | 9.27 |
| Pharmacology | 82 | 2.00 |
| CHEMISTRY (general) | 77 | 1.87 |
| Analytical chemistry | 88 | 2.14 |
| Inorganic chemistry | 132 | 3.21 |
| Medicinal/pharmaceutical chemistry | 30 | 0.73 |
| Organic chemistry | 195 | 4.75 |
| Physical chemistry | 205 | 4.99 |
| PHYSICS (general) | 127 | 3.09 |
| Astronomy/astrophysics | 127 | 3.09 |
| Biophysics | 90 | 2.19 |
| Condensed matter/low-temperature physics | 233 | 5.67 |
| Optics/photonics | 79 | 1.92 |
| Nuclear physics | 62 | 1.51 |
| Particle physics | 176 | 4.28 |
| Applied physics | 65 | 1.58 |
| **Total** | **4,109** | **100.00** |
