# Supplementary material for: Science PhD Career Preferences: Levels, Changes, and Advisor Encouragement
Source: PLoS One. 2012 May 2;7(5):e36307. doi: 10.1371/journal.pone.0036307 (PMC3342243; doi:10.1371/journal.pone.0036307)
Supplement: Table S3 — Summary statistics, by field. (DOCX) [file pone.0036307.s003.docx]

Table S3: Summary statistics, by field

|  |  | **Bio/Life** | | **Chemistry** | | **Physics** | |
| --- | --- | --- | --- | --- | --- | --- | --- |
| **Question block** | **Variable** | **Mean** | **Std. Dev.** | **Mean** | **Std. Dev.** | **Mean** | **Std. Dev.** |
| Current career preferences | faculty - teaching | 3.61 | 1.14 | 3.51 | 1.18 | 3.63 | 1.14 |
|  | faculty - research | 3.83 | 1.17 | 3.34 | 1.25 | 4.02 | 1.04 |
|  | government | 3.75 | 0.93 | 3.79 | 0.94 | 3.82 | 0.91 |
|  | established firm | 3.53 | 1.05 | 3.83 | 1.02 | 3.61 | 0.97 |
|  | startup firm | 3.25 | 1.05 | 3.44 | 1.03 | 3.37 | 1.02 |
|  |  |  |  |  |  |  |  |
| Stage in the PhD program | stage: early | 0.27 |  | 0.26 |  | 0.24 |  |
|  | stage: middle | 0.35 |  | 0.38 |  | 0.44 |  |
|  | stage: late | 0.38 |  | 0.36 |  | 0.32 |  |
|  |  |  |  |  |  |  |  |
| Career preferences at start of PhD program | faculty - teaching | 3.25 | 1.08 | 3.19 | 1.19 | 3.31 | 1.03 |
|  | faculty - research | 3.66 | 1.04 | 3.17 | 1.19 | 3.77 | 0.97 |
|  | government | 3.07 | 0.96 | 3.24 | 1.00 | 3.22 | 0.95 |
|  | established firm | 3.04 | 1.09 | 3.45 | 1.06 | 3.16 | 1.00 |
|  | startup firm | 2.72 | 1.07 | 3.05 | 1.05 | 2.83 | 1.02 |
|  |  |  |  |  |  |  |  |
| Interest in work activities | basic research | 4.19 | 0.83 | 3.85 | 0.95 | 4.26 | 0.84 |
|  | applied research | 4.31 | 0.69 | 4.37 | 0.65 | 4.19 | 0.70 |
|  | development | 3.23 | 1.06 | 3.77 | 0.97 | 3.62 | 0.96 |
|  | commercialization | 3.09 | 1.13 | 3.43 | 1.07 | 3.00 | 1.13 |
|  | management/administration | 2.79 | 1.16 | 3.02 | 1.14 | 2.57 | 1.13 |
|  | teaching | 3.89 | 0.93 | 3.79 | 0.95 | 3.73 | 0.94 |
|  |  |  |  |  |  |  |  |
| Degree to which careers are encouraged/discouraged in lab/department | faculty - teaching | 3.60 | 0.80 | 3.58 | 0.75 | 3.53 | 0.72 |
|  | faculty - research | 4.36 | 0.72 | 4.13 | 0.73 | 4.07 | 0.78 |
|  | government | 3.39 | 0.69 | 3.60 | 0.67 | 3.58 | 0.71 |
|  | established firm | 3.23 | 0.73 | 3.69 | 0.69 | 3.30 | 0.67 |
|  | startup firm | 3.13 | 0.71 | 3.47 | 0.69 | 3.20 | 0.64 |
