# Supplementary material for: Science PhD Career Preferences: Levels, Changes, and Advisor Encouragement
Source: PLoS One. 2012 May 2;7(5):e36307. doi: 10.1371/journal.pone.0036307 (PMC3342243; doi:10.1371/journal.pone.0036307)
Supplement: Table S4 — Detailed distribution of current career preferences, by stage in program. (DOCX) [file pone.0036307.s004.docx]

Table S4: Detailed distribution of current career preferences, by stage in program

|  |  | faculty - teaching | | faculty - research | | government | | established firm | | startup firm | |
| --- | --- | --- | --- | --- | --- | --- | --- | --- | --- | --- | --- |
|  |  | Early | Late | Early | Late | Early | Late | Early | Late | Early | Late |
|  |  |  |  |  |  |  |  |  |  |  |  |
| Bio/Life | Extremely unattractive | 4% | 6% | 2% | 6% | 2% | 3% | 4% | 5% | 4% | 7% |
|  | Unattractive | 15% | 17% | 9% | 15% | 8% | 8% | 11% | 13% | 17% | 18% |
|  | Neither attractive nor unattractive | 18% | 18% | 11% | 12% | 19% | 20% | 27% | 24% | 33% | 28% |
|  | Attractive | 39% | 39% | 39% | 34% | 51% | 50% | 41% | 42% | 37% | 37% |
|  | Extremely attractive | 25% | 21% | 39% | 33% | 20% | 19% | 18% | 16% | 8% | 10% |
|  | Total | 100% | 100% | 100% | 100% | 100% | 100% | 100% | 100% | 100% | 100% |
|  |  |  |  |  |  |  |  |  |  |  |  |
| Chemistry | Extremely unattractive | 5% | 7% | 6% | 10% | 1% | 2% | 2% | 2% | 3% | 3% |
|  | Unattractive | 17% | 17% | 16% | 28% | 6% | 8% | 10% | 9% | 14% | 15% |
|  | Neither attractive nor unattractive | 16% | 19% | 16% | 15% | 18% | 17% | 18% | 13% | 31% | 24% |
|  | Attractive | 41% | 40% | 40% | 26% | 51% | 48% | 47% | 39% | 41% | 41% |
|  | Extremely attractive | 21% | 16% | 22% | 21% | 24% | 24% | 23% | 37% | 11% | 17% |
|  | Total | 100% | 100% | 100% | 100% | 100% | 100% | 100% | 100% | 100% | 100% |
|  |  |  |  |  |  |  |  |  |  |  |  |
| Physics | Extremely unattractive | 5% | 5% | 2% | 4% | 1% | 2% | 4% | 4% | 4% | 6% |
|  | Unattractive | 15% | 15% | 5% | 10% | 5% | 8% | 11% | 8% | 17% | 14% |
|  | Neither attractive nor unattractive | 16% | 19% | 11% | 14% | 18% | 19% | 19% | 23% | 28% | 28% |
|  | Attractive | 40% | 38% | 44% | 36% | 53% | 50% | 54% | 50% | 42% | 38% |
|  | Extremely attractive | 24% | 23% | 37% | 36% | 23% | 21% | 12% | 15% | 9% | 13% |
|  | Total | 100% | 100% | 100% | 100% | 100% | 100% | 100% | 100% | 100% | 100% |
