# Supplementary material for: Science PhD Career Preferences: Levels, Changes, and Advisor Encouragement
Source: PLoS One. 2012 May 2;7(5):e36307. doi: 10.1371/journal.pone.0036307 (PMC3342243; doi:10.1371/journal.pone.0036307)
Supplement: Table S5 — Data for Figure 4 (share of students reporting that particular careers are encouraged/discouraged in their lab or department). (DOCX) [file pone.0036307.s005.docx]

Table S5: Data for Figure 4 (share of students reporting that particular careers are encouraged/discouraged in their lab or department)

|  | **Bio/Life** | | | | |
| --- | --- | --- | --- | --- | --- |
|  | faculty - teaching | faculty - research | government | established firm | startup firm |
| Strongly discouraged | 1% | 0% | 1% | 2% | 2% |
| Discouraged | 6% | 0% | 4% | 9% | 9% |
| Neither encouraged nor discouraged | 36% | 14% | 56% | 58% | 64% |
| Encouraged | 46% | 36% | 34% | 27% | 22% |
| Strongly encouraged | 11% | 50% | 5% | 4% | 3% |
|  |  |  |  |  |  |
|  | **Chemistry** | | | | |
|  | faculty - teaching | faculty - research | government | established firm | startup firm |
| Strongly discouraged | 0% | 0% | 0% | 0% | 1% |
| Discouraged | 6% | 0% | 2% | 3% | 4% |
| Neither encouraged nor discouraged | 38% | 19% | 44% | 35% | 49% |
| Encouraged | 46% | 47% | 46% | 52% | 41% |
| Strongly encouraged | 9% | 33% | 8% | 10% | 6% |
|  |  |  |  |  |  |
|  | **Physics** | | | | |
|  | faculty - teaching | faculty - research | government | established firm | startup firm |
| Strongly discouraged | 0% | 0% | 0% | 1% | 1% |
| Discouraged | 5% | 1% | 2% | 6% | 6% |
| Neither encouraged nor discouraged | 45% | 24% | 47% | 59% | 68% |
| Encouraged | 41% | 42% | 41% | 31% | 22% |
| Strongly encouraged | 8% | 33% | 10% | 3% | 3% |
