# Supplementary material for: Science PhD Career Preferences: Levels, Changes, and Advisor Encouragement
Source: PLoS One. 2012 May 2;7(5):e36307. doi: 10.1371/journal.pone.0036307 (PMC3342243; doi:10.1371/journal.pone.0036307)
Supplement: Table S6 — Data for Figure 5 (share of students finding particular work activities interesting/uninteresting). (DOCX) [file pone.0036307.s006.docx]

Table S6: Data for Figure 5 (share of students finding particular work activities interesting/uninteresting)

|  | **Bio/Life** | | | | | |
| --- | --- | --- | --- | --- | --- | --- |
|  | Basic R. | Applied R. | Development | Commercialization | Management/Admin | Teaching |
| Extremely uninteresting | 1% | 0% | 6% | 9% | 16% | 2% |
| Uninteresting | 4% | 2% | 20% | 23% | 26% | 7% |
| Neither interesting nor uninteresting | 8% | 7% | 30% | 28% | 28% | 16% |
| Interesting | 48% | 50% | 35% | 31% | 24% | 48% |
| Extremely interesting | 39% | 41% | 10% | 10% | 6% | 26% |
|  |  |  |  |  |  |  |
|  | **Chemistry** | | | | | |
|  | Basic R. | Applied R. | Development | Commercialization | Management/Admin | Teaching |
| Extremely uninteresting | 2% | 0% | 2% | 4% | 11% | 2% |
| Uninteresting | 9% | 1% | 10% | 17% | 22% | 8% |
| Neither interesting nor uninteresting | 13% | 5% | 20% | 25% | 29% | 21% |
| Interesting | 53% | 49% | 45% | 39% | 29% | 47% |
| Extremely interesting | 23% | 45% | 23% | 15% | 9% | 22% |
|  |  |  |  |  |  |  |
|  | **Physics** | | | | | |
|  | Basic R. | Applied R. | Development | Commercialization | Management/Admin | Teaching |
| Extremely uninteresting | 1% | 1% | 3% | 11% | 21% | 2% |
| Uninteresting | 4% | 3% | 12% | 23% | 29% | 8% |
| Neither interesting nor uninteresting | 8% | 6% | 22% | 29% | 26% | 22% |
| Interesting | 42% | 59% | 49% | 30% | 21% | 49% |
| Extremely interesting | 45% | 32% | 15% | 8% | 3% | 19% |
